# Effects of multiple object tracking training on visual attention and sports decision-making in novice female basketball players: evidence from EyeLink-EEG synchronization

**DOI:** 10.7717/peerj.21711

**Published:** 2026-09-09

**Authors:** Qifeng Gou, Yongfeng Liu, Sunnan Li

**Affiliations:** 1 College of Sports Training, Chengdu Sport University, Chengdu, Sichuan, China; 2 College of Physical Education, Northwest Normal University, Lanzhou, Gan Su, China; 3 College of P.E. and Sports, Beijing Normal University, Beijing, China

**Keywords:** Multiple object tracking, Female basketball player, Sports decision-making, Eyelink-EEG synchronization

## Abstract

**Background:**

Visual attention is critical for effective sports decision-making (SDM), this study explores that how multiple-object tracking (MOT) training improves decision-making accuracy (ACC) and the underlying neural mechanisms.

**Methods:**

The paper selected 56 novice female novice basketball players from universities, which synchronized eye movement electroencephalogram (EEG) technology was used to analyze the relationship between visual attention and sports decision-making, as well as the corresponding EEG changes, through nine weeks of multiple-object tracking training in the intervention group.

**Results:**

After training, the accuracy of the intervention group significantly improved, the proportion of players’ fixation on key and related areas increased, and the visual search process was simplified. During the decision-making process, event-related potential (ERP) components N1, P1, late positive complex (LPC), and late negative complex (LNC) undergo changes, the result improved efficiency of cognitive processing for players.

**Conclusions:**

Nine weeks multiple-object tracking training can improve the sports decision-making ability of novice female basketball players significantly. After training, the proportion of fixation in the key and related areas of players increased. During the decision-making process, the ERP components N1, P1, LPC decreased, and LNC increased, the result indicates that more cognitive resources were allocated effectively to the key information and optimal time points for completing decisions, demonstrating cognitive processing advantages.

## Introduction

Sports decision-making refers to the process in which players evaluate available information and weigh pros and cons in a short period of time to make an action plan under complex sports scenarios (Ren, 2019). In players’ performance, decision-making ability is critical for achieving excellence in competition. The integration of accurate decision-making and advanced technical skills were considered one of the key factors for outstanding performance (Marasso et al., 2014), especially more than 80% of brain information is derived from the visual system (Sui, Gao & Xiang, 2018). Previous studies have shown that expert players, who due to long-term specialized training, which possess superior strategies for processing action-related information, demonstrating a stronger ability to identify and process relevant cues in sports situations at early stages (De Assis et al., 2021).

However, existing literature has largely emphasized descriptive differences between expert and novice performers, while offering limited theoretical integration regarding the mechanisms through which perceptual–cognitive training enhances sports decision-making. Moreover, prior studies tend to examine isolated components such as visual search or reaction time (RT), without systematically linking these processes to trainable cognitive interventions. Although attention training is regarded as essential in sports instruction widely, research focuses on developing players’ attentional abilities from a sports-cognitive perspective remains limited (Jin, 2020).

This gap highlights the need for research that not only examines perceptual–cognitive abilities but also evaluates targeted training paradigms capable of improving these abilities and transferring them to real-game decision-making.

Multiple-object tracking (MOT) is expected to enhance sports decision-making (SDM) because it targets core perceptual–cognitive processes essential for team sports. MOT trains dynamic selective attention, which divided attention and the ability to monitor multiple moving elements—abilities directly required when players track teammates, opponents, and spatial changes during play. Neurophysiological evidence shows that MOT activates the fronto-parietal attention network (Faubert, 2013),which overlaps with brain regions supporting anticipation and action selection. Moreover, expert-novice research indicates that superior SDM relies on efficient visual search and cue to extraction (Mann et al., 2007). Nevertheless, despite these theoretical connections, empirical evidence directly linking MOT training to improvements in sports-specific decision-making—particularly in ecologically valid contexts—remains limited and sometimes inconsistent across studies. Additionally, few studies have examined the neural mechanisms underlying such potential transfer effects, leaving the cognitive and neurophysiological pathways insufficiently understood. Thus, MOT provides both perceptual demands and neural activation patterns that support transfer to improved SDM theoretically.

Basketball represents a highly dynamic and cognitively demanding sport that places substantial demands on visual attention and rapid decision-making. Players are required to continuously monitor teammates and opponents while adapting to rapidly chang spatial configurations. Consequently, enhancing attentional capacity to improve decision-making accuracy has become a key concern for both researchers and practitioners (Jin, 2020). While prior studies have contributed to the understanding of sports decision-making significantly, many of which have suffered from limited ecological validity. Most existing research presents stimuli from a third-person perspective (Li, Xu & Zhang, 2006), despite the substantial differences in information processing between first-person and third-person views. Such methodological limitations restrict the generalizability of findings, as third-person perspectives fail to accurately replicate the perceptual–motor coupling inherent in actual gameplay. The first-person perspective provides clearer representations of reachable objects and offers more precise information regarding object identity and physical features, which may enhance action prediction and judgment (Zhao, 2020). Despite this advantage, the integration of first-person paradigms with perceptual–cognitive training interventions remains underexplored.

Furthermore, although visual attention has been identified as a key factor influencing sports decision-making, existing studies rarely adopt multimodal approaches to simultaneously capture behavioral, visual, and neural data. This lack of methodological integration limits a comprehensive understanding of how attentional training influences decision-making processes at both behavioral and neurophysiological levels.

This study employs a combination of eye-tracking and electroencephalogram (EEG) techniques to reflect the physiological processes of information processing in the brain synchronously. Eye-tracking was recognized as one of the most effective methods in the study of visual information processing, and allowing researchers to capture real-time visual behavior during sports tasks-something that is difficult to achieve through other methodologies (Li, 2015). Meanwhile, EEG offers high temporal resolution for identifying neural dynamics associated with cognitive processing stages, enabling a more detailed examination of decision-making mechanisms.

Based on prior theoretical and empirical evidence, the following hypotheses were proposed:

H1: MOT training will improve decision-making performance significantly, reflected in shorter reaction time and higher accuracy, particularly in cognitively demanding decision conditions.

H2: MOT training will optimize visual search behavior, which characterized by shorter fixation durations, fewer fixations, and greater allocation of gaze to task-relevant areas.

H3: MOT training will modulate event-related potential (ERP) components associated with attentional allocation and cognitive processing (*e.g.*, P1, N1, late positive complex (LPC), and late negative complex (LNC)), indicating enhanced neural efficiency during decision-making.

Through experimental intervention, this study investigates the impact of attentional training on the decision-making abilities of novice basketball players and explores the underlying neural mechanisms. By integrating perceptual training, ecologically valid task design, and multimodal measurement, the present study aims to provide a more comprehensive understanding of the transfer effects of attentional training on sports decision-making. The findings aim to provide both theoretical support and practical implications for enhancing the quality of basketball teaching and training.

## Materials & Methods

### Overall methodological pipeline

**Figure 1:**
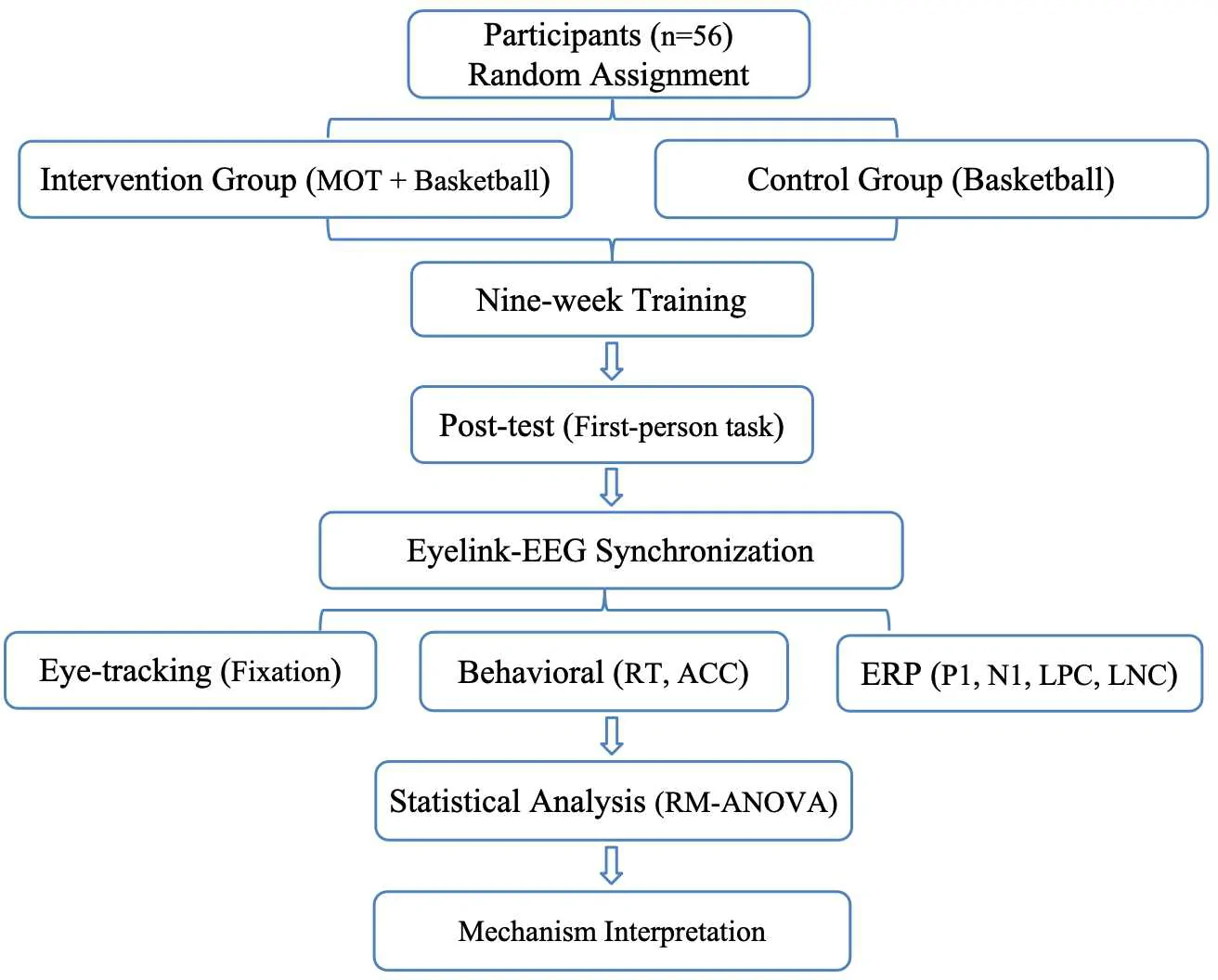
Overall methodological pipeline of the study.

The overall methodological pipeline of the present study is illustrated in Fig. 1. The experimental procedure consisted of four main stages:

Stage 1: Participant allocation and baseline assessment. Participants were assigned randomly to the intervention group and control group, and pre-test measurements of behavioral performance, eye movements, and EEG signals were conducted.

Stage 2: Intervention phase. The intervention group underwent a nine-week MOT training program in addition to regular basketball instruction, while the control group only received standard basketball training.

Stage 3: Post-test and multimodal data acquisition. After the intervention, both groups completed the same decision-making tasks under synchronized eye-tracking and EEG recording conditions. Behavioral (reaction time, accuracy), visual (fixation duration, fixation count), and neural (ERP components) data were collected simultaneously.

Stage 4: Data processing and statistical analysis. Behavioral, eye-tracking, and EEG data were preprocessed and analyzed using repeated measures analysis of variance (ANOVA) to examine the effects of training, decision type, and task conditions.

This integrated pipeline enables a comprehensive examination of the perceptual–cognitive and neural mechanisms underlying sports decision-making.

### Participants

The sample size for this study was estimated using G*Power 3.1.9.7 software. Assuming a medium effect size (*f* = 0.25) and an alpha level of 0.05, a total of 48 participants (24 per group) would be required to achieve a statistical power of 0.80 (Lakens, 2014). *A priori* power analysis was conducted in accordance with established methodological recommendations rather than derived from a single empirical MOT study reporting a three-way interaction. To account for potential dropouts, 56 participants were recruited and assigned randomly to either the intervention group or the control group, with 28 participants in each group. All participants were female students enrolled in a basketball elective course at Northwest Normal University. The intervention group had a mean age of 21.00 ± 0.72 years and an average training experience of 1.25 ± 0.32 years. The control group had a mean age of 20.00 ± 0.85 years and an average training experience of 1.50 ± 0.31 years. An independent samples *t*-test conducted at pre-test indicated no significant difference in training experience between the intervention and control groups (*p* > 0.05), suggesting baseline equivalence. None of the participants held an official athletic classification, all were right-handed, and all had normal or corrected-to-normal vision.

The study was approved by the Ethics Committee of Northwest Normal University (No. NWNU-20230301). Prior to the experiment, participants were fully informed of the study’s purpose and procedures and signed written informed consent forms.

### MOT training

Participants in the intervention group were allowed to use their own laptops to complete the training. To ensure consistency across devices, all laptops met standardized specifications (Windows 10, ≥2.4 GHz CPU, 15.6-inch display, 1,920 × 1,080 resolution, 60 Hz refresh rate), which guaranteed uniform stimulus presentation and minimized potential variability due to hardware differences. The study used a self-developed MOT training software (Registration No. 2023SR0414381), which included adjustable settings for the number of targets (2, 3, 4, 5, or 6) and target speeds (5°/s, 10°/s, 15°/s), which allowing students to train at various difficulty levels (Liu, 2019). The theoretical basis for including the number of targets in four or more is not to assume infinite tracking ability, but to systematically manipulate task difficulty and attention load over a larger range. Pilot testing confirmed that participants were able to perceive and track targets accurately across all speed conditions, with which no subjective reports of excessive blur or perceptual difficulty attributable to display limitations.

### MOT training protocol

The intervention was conducted from March 1 to May 3, 2023, which spanning a total of 9 weeks. Both the intervention and control groups participated in regular basketball instruction, consisting of two class hours per week for a total of 18 sessions. In addition, the intervention group engaged in 20-minute MOT training sessions at the end of each class and completed two additional 20-minute sessions per week, scheduled every other day, totaling three training sessions per week.

### Design

A 2 (group: intervention, control) × 2 (decision type: intuitive, cognitive) × 3 (offensive strategy: passing, shooting, breakthrough) × 2 (test time: pre-test, Post-test) four-factor mixed experimental design was employed. Group was treated as the between-subjects variable, while decision type, offensive strategy, and test time were within-subjects variables. The dependent variables included reaction time, decision accuracy (number of correct responses / total responses), and average fixation duration. Repeated measures ANOVA were used for data analysis.

### Stimuli

Stimuli were selected from frontcourt offensive plays in Women’s Chinese Basketball Association (WCBA) league games. All video clips were finalized through discussion with basketball coaches and players and were filmed from a first-person perspective to better replicate the actual decision-making context (Ping, 2019). (1) The selection of stimulating materials: ① Whether it is a successful offensive game scene? ② Is the number of people in the screen sufficient (usually requiring the same screen to include both offensive and defensive players, with at least five people)? ③ Are the team members’ movements clear? (2) Validity of stimulus materials

① Determination of content validity

Content validity is an important indicator for measuring the scientificity and applicability of experimental materials, which used to ensure that the experimental materials can reflect the specific tasks or behaviors of the research subjects comprehensively and accurately. Based on the content validity ratio (CVR) and content validity index (CVI) methods proposed by Lawshe (1975), combined with the commonly used expert evaluation method in sports psychology research. This study systematically verifies the content validity of video materials. The final first-person perspective attack video was shot from the selected 129 WCBA league forward position attack videos, and the content validity is determined by scoring and evaluation of five basketball experts.

② Determination of structural validity

The study also selected six high-level basketball players and six basketball elective college students for validity testing, which aiming to eliminate the “ceiling effect” and “floor effect” and ensure that each video stimulus material can effectively distinguish the decision-making efficiency of athletes at different levels. For videos with low discrimination, the video duration can be extended or shortened for re editing until each video can distinguish effectively the differences in decision-making agility and rationality between high-level athletes and elective course students, that means, high-level athletes have higher decision-making speed and accuracy, thus ultimately forming the experimental materials for this study. The final editing time for the video material is 3,000 ms.

A total of 129 video clips were used in this study, including nine for practice and 120 for formal testing—comprising 40 clips each for passing, shooting, and breakthrough decisions. The number of videos assigned to cognitive and intuitive decision-making tasks were equal (Zhang, 2021), which ensurs a balanced probability distribution across the three types of offensive decisions.

### Procedures

**Figure 2:**
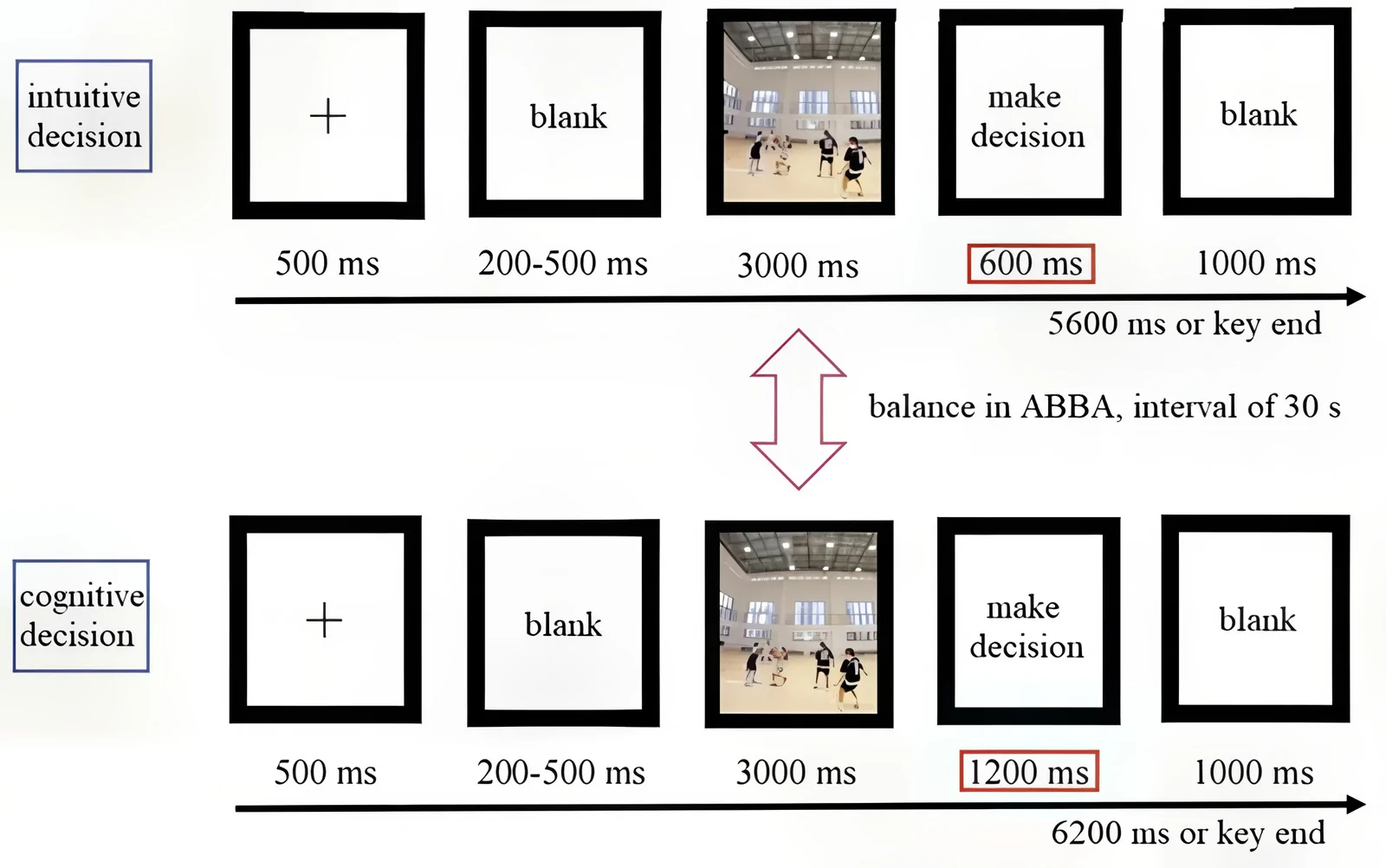
Basic process of Eyelink-EEG synchronization experiment.

The experimental procedure is shown in Fig. 2.

### Eye-tracking measurement

**Figure 3:**
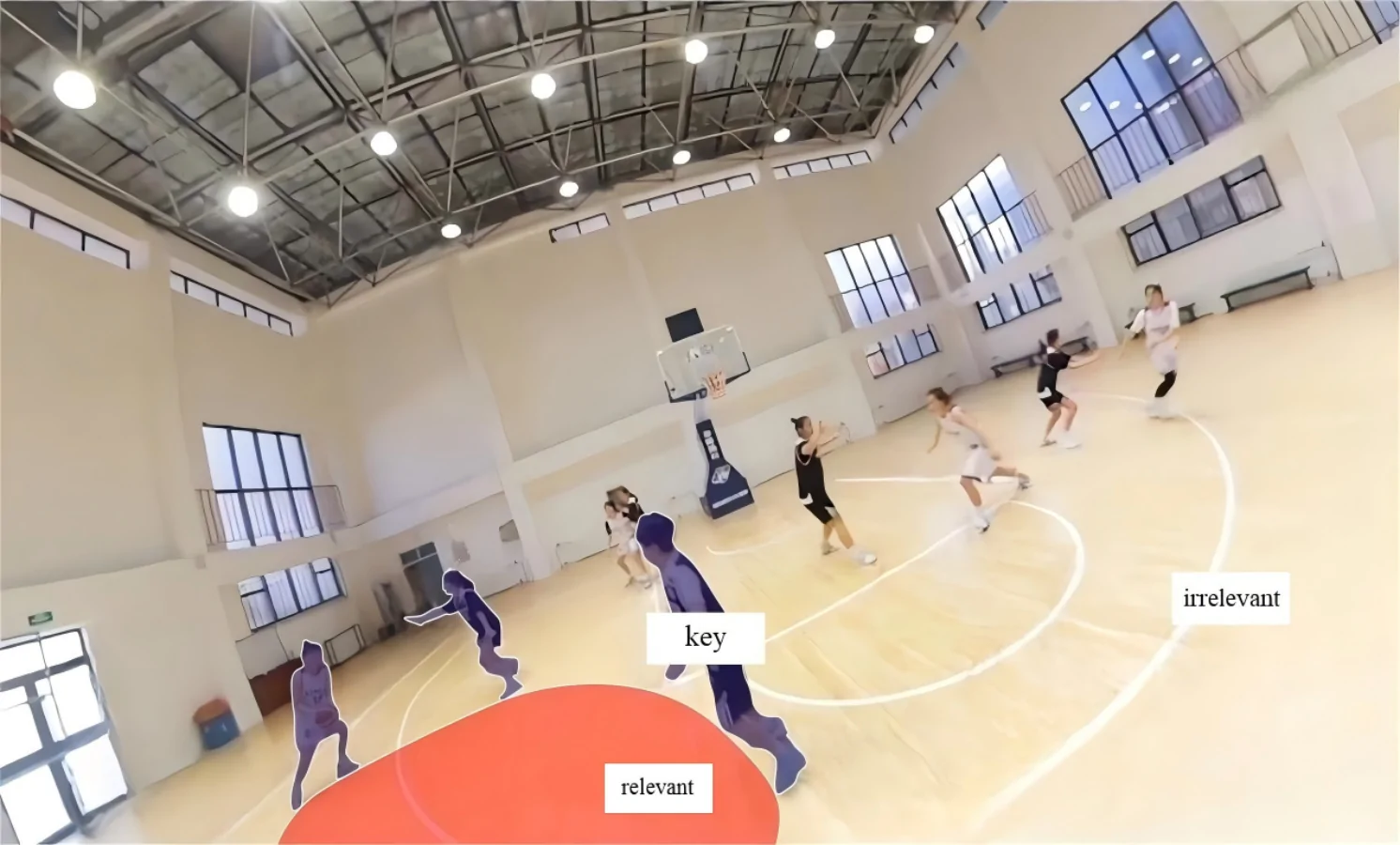
Legend of interest area editing.

The eye-tracking component of the experiment was conducted using the Eyelink eye-tracking data acquisition and analysis system developed by SR Research Ltd. (Canada). An Eyelink Portable Duo device was employed, with which participants’ head movements stabilized using a chinrest, and eye movement data recorded at a sampling rate of 1,000 Hz. For video-based dynamic stimuli, dynamic Areas of Interest (AOIs) were manually created frame-by-frame using a mouse. Based on previous literature and expert consultations—including two basketball coaches, one active CBA player, and one Eyelink engineer—the AOIs were categorized into three types: key AOIs, relevant AOIs, and irrelevant AOIs (Jin, 2020). Key AOIs were defined as regions directly associated with the decision outcome (*e.g.*, ball carrier, primary defender, intended target, or basket), relevant AOIs included contextual elements such as nearby players and open space, and irrelevant AOIs referred to regions with minimal decision-related information. AOIs were dynamically defined frame-by-frame based on the spatial positions of players, the ball, and the basket. The legend of the interest area editing is shown in Fig. 3.

### EEG measurement

EEG data were collected using a 64-channel EEG acquisition system (ANT Neuro, The Netherlands), with electrodes arranged according to the extended International 10–20 system. Horizontal electrooculograms (HEOGs) were recorded using electrodes placed one cm from the outer canthi of both eyes, and vertical electrooculograms (VEOGs) were recorded using an electrode placed one cm below the left eye. Data from the mastoids (M1 and M2) were also recorded. The sampling rate was 1,000 Hz per channel. CPz served as the online reference electrode, and the GND point was used for grounding. Electrode impedance was maintained below five kΩ throughout data collection (Jia et al., 2022).

### Data collection and analysis

Behavioral data were collected using E-Prime 3.0 software. Data preprocessing—including filtering, merging, and excluding outliers beyond ±3 standard deviations from the mean—was performed using E-Data Aid. The behavioral indicators analyzed including reaction time and decision accuracy. Eye-tracking data were processed and analyzed using Data Viewer 4.2 software and subsequently exported in Excel format for further statistical analysis.

SPSS 29.0 software was used to conduct repeated measures ANOVA, with behavioral indicators, eye-tracking metrics, and ERP components set as dependent variables. A 2 (group: intervention, control) × 2 (decision type: intuitive, cognitive) × 3 (offensive strategy: passing, shooting, breakthrough) × 2 (test time: pre-test, Post-test) design was applied. Where significant interaction effects were found, simple effects analyses were conducted. *Post hoc* comparisons were performed using the Bonferroni correction. A significance level of *P* < 0.05 was adopted as the criterion for statistical significance.

Ensuring clock synchronization between the two data acquisition computers during real-time collection of eye-tracking and EEG data is crucial (Xue, 2017), A TTL signal was sent to the ANT Neuro amplifier *via* a parallel port, while a corresponding message was simultaneously transmitted to the Eyelink Host PC *via* Ethernet. This combination of TTL + message markers was used to align the eye-tracking and EEG datasets during the analysis phase. Six categories of stimuli were marked using numeric codes 1–6: cognitive-passing, cognitive-shooting, cognitive-breakthrough, intuitive-passing, intuitive-shooting, and intuitive-breakthrough. Additional event codes 1–5 were used to indicate the following behavioral responses: video start, video end, correct response, incorrect response, and no response. For example, the onset of an intuitive-breakthrough video was marked as 61. EEG data were processed using the EEGLAB toolbox in MATLAB. Firstly, the raw 62-channel EEG data were preprocessed to remove abnormal epochs caused by line noise or head movement artifacts. Baseline correction was applied to remove drift, the sampling rate was downsampled to 500 Hz, and an average reference was computed across all electrodes. A 0.5–40 Hz bandpass filter was applied, and Independent Component Analysis (ICA) was used to eliminate ocular artifacts. Additional artifacts exceeding ±100 µV in amplitude were also excluded from analysis. Event-related EEG epochs were then extracted from the preprocessed data based on the designated markers-for example, epoch11, epoch21, epoch31, epoch41, epoch51, and epoch61 corresponded to video onset events for each of the six stimulus types. ERP waveforms were computed using a 200 ms pre-stimulus baseline. The following analyses were conducted (Liu, 2016): (1) Early component analysis: Using video onset as time zero, the time window of 0–1,000 ms post-stimulus was analyzed to examine ERP waveforms, topographic maps, and amplitudes of early components. (2) Late component analysis: Using video onset as time zero again, the 1,000–3,000 ms post-stimulus window was divided into multiple shorter time segments. Within each segment, the average ERP amplitudes at relevant electrode sites were computed to observe late components. (3) Post-video offset analysis: Using video offset as time zero, the 0–600 ms post-offset window was analyzed to assess ERP waveforms, topographic distributions, and amplitude characteristics associated with the termination of visual stimuli.

## Results

### Comparison of decision-making reaction time between the intervention and control groups

A repeated measures ANOVA was conducted with group (intervention group, control group) as the between-subjects variable, and decision type (intuitive, cognitive), offensive strategy (passing, shooting, breakthrough), and test time (pre-test, Post-test) as within-subjects variables. The dependent variable was reaction time. The results are presented in Table 1.

**Table 1:** Comparison of RT between intervention group and control group.

Group	Type	Time	Passing	Shooting	Breakthrough	Repeated measures ANOVA		
			*M*±*SD*	*M*±*SD*	*M*±*SD*	*F*	*P*	*η* ^2^
Intervention	Intuitive	Pre-test	420.38 ± 119.63	448.45 ± 112.07	425.04 ± 119.82			
			Post-test	380.39 ± 126.22	469.41 ± 108.91	418.93 ± 111.33			
		Cognitive	Pre-test	624.32 ± 205.16	776.48 ± 171.90	732.30 ± 157.67			
	Post-test		509.56 ± 214.80	668.14 ± 180.53	709.62 ± 196.28			
Control	Intuitive	Pre-test	425.11 ± 110.51	409.56 ± 141.11	415.18 ± 143.36			
			Post-test	393.33 ± 130.87	493.50 ± 93.33	465.27 ± 102.83			
		Cognitive	Pre-test	686.39 ± 212.82	706.78 ± 259.30	749.96 ± 196.71			
	Post-test		618.56 ± 222.46	639.21 ± 176.48	715.23 ± 202.04			
Group						0.280	0.599	0.005
Type						483.654	<0.001^**^	0.900
Group × Type						0.174	0.678	0.003
Strategy						9.788	<0.001^**^	0.153
Group × Strategy						2.124	0.124	0.038
Time						5.595	0.022^*^	0.094
Group × Time						2.010	0.162	0.036
Type × Strategy						7.828	<0.001^**^	0.127
Group × Type × Strategy						3.489	0.034^*^	0.061
Type × Time						16.189	<0.001^**^	0.231
Group × Type × Time						0.178	0.675	0.003
Strategy × Time						1.945	0.148	0.035
Group × Strategy × Time						0.124	0.884	0.002
Type × Strategy × Time						2.627	0.077	0.046
Group × Type × Strategy × Time						0.742	0.479	0.014

**Notes.**

reaction time (ms)

* *P* < 0.05

** *P* < 0.01

The results showed a significant main effect of decision type, with intuitive decisions (430.38 ± 118.33 ms) exhibiting significantly shorter reaction time than cognitive decisions (678.05 ± 199.68 ms), *F*(1, 54) = 483.654, *P* < 0.001, *η*^2^ = 0.900. A significant main effect of offensive strategy was also observed, *F*(2, 108) = 9.788, *P* < 0.001, *η*^2^ = 0.153. *Post hoc* tests indicated that reaction time for shooting and breakthrough were significantly longer than for passing (*Ps* < 0.01), while there was no significant difference between shooting and breakthrough (*P* > 0.05). A significant main effect of test time was found, with Post-test reaction time (540.10 ± 155.51 ms) being significantly shorter than pre-test (568.33 ± 162.51 ms), *F*(1, 54) = 5.595, *P* = 0.022, *η*^2^ = 0.094. The interaction effect between decision type and offensive strategy was significant, *F*(2, 108) = 7.828, *P* < 0.001, *η*^2^ = 0.127. The three-way interaction between group, decision type, and offensive strategy was also significant, *F*(2, 108) = 3.489, *P* = 0.034, *η*^2^ = 0.061. A significant interaction between decision type and test time was observed, *F*(1, 54) = 16.186, *P* < 0.001, *η*^2^ = 0.231. No other significant main or interaction effects were found (*Ps* > 0.05).

To further examine the interaction between decision type and offensive strategy, which simply effects analyses revealed that, for all three offensive strategies (passing, shooting, breakthrough), the reaction time for cognitive decisions was significantly longer than for intuitive decisions (*Ps* < 0.001).

To further explore the three-way interaction among group, decision type, and offensive strategy, simple effects analyses showed that, under intuitive decision conditions, there were no significant differences in reaction time between the intervention and control groups across all offensive strategies (*Ps* > 0.05). Under cognitive decision conditions, the reaction time for passing in the intervention group was marginally shorter than that in the control group (*P* = 0.073), while no significant differences were found in shooting and breakthrough (*Ps* > 0.05).

To further examine the interaction between decision type and test time, which simply effects analyses revealed that there was no significant difference in reaction time for intuitive decisions between pre-test (423.95 ± 124.42 ms) and Post-test (436.81 ± 112.25 ms), *P* = 0.125. However, for cognitive decisions, the Post-test reaction time (643.39 ± 198.76 ms) was significantly shorter than the pre-test (712.70 ± 200.59 ms), *P* = 0.001.

### Comparison of decision accuracy between the intervention and control groups

A repeated measures ANOVA was conducted with group (intervention group, control group) as the between-subjects variable, and decision type (intuitive, cognitive), offensive strategy (passing, shooting, breakthrough), and test time (pre-test, Post-test) as within-subjects variables. The dependent variable was decision accuracy. The results are presented in Table 2.

**Table 2:** Comparison of accuracy (ACC) between intervention group and control group.

Group	Type	Time	Passing	Shooting	Breakthrough	Repeated measures ANOVA		
			*M*±*SD*	*M*±*SD*	*M*±*SD*	*F*	*P*	*η* ^2^
Intervention	Intuitive	Pre-test	0.34 ± 0.11	0.36 ± 0.13	0.32 ± 0.08			
			Post-test	0.42 ± 0.12	0.35 ± 0.09	0.32 ± 0.10			
		Cognitive	Pre-test	0.31 ± 0.13	0.43 ± 0.12	0.39 ± 0.07			
	Post-test		0.55 ± 0.11	0.47 ± 0.10	0.40 ± 0.10			
Control	Intuitive	Pre-test	0.33 ± 0.12	0.36 ± 0.10	0.32 ± 0.14			
			Post-test	0.35 ± 0.13	0.34 ± 0.11	0.30 ± 0.11			
		Cognitive	Pre-test	0.32 ± 0.14	0.44 ± 0.09	0.41 ± 0.12			
	Post-test		0.33 ± 0.16	0.48 ± 0.09	0.40 ± 0.09			
Group						2.064	0.157	0.037
Type						119.504	<0.001^**^	0.689
Group × Type						0.470	0.496	0.009
Strategy						5.672	0.005^**^	0.095
Group × Strategy						4.657	0.011^*^	0.079
Time						30.035	<0.001^**^	0.357
Group × Time						30.148	<0.001^**^	0.358
Type × Strategy						16.119	<0.001^**^	0.230
Group × Type × Strategy						4.409	0.014^*^	0.075
Type × Time						10.135	0.002^**^	0.160
Group × Type × Time						4.534	0.038^*^	0.077
Strategy × Time						25.219	<0.001^**^	0.318
Group × Strategy × Time						13.809	<0.001^**^	0.204
Type × Strategy × Time						3.136	0.047^*^	0.055
Group × Type × Strategy × Time						4.679	0.011^*^	0.080

**Notes.**

* *P* < 0.05

** *P* < 0.01

The results revealed a significant main effect of decision type, with the accuracy of intuitive decisions (0.34 ± 0.11) significantly lower than that of cognitive decisions (0.41 ± 0.11), *F*(1, 54) = 119.504, *P* < 0.001, *η*^2^ = 0.689. A significant main effect of offensive strategy was found, *F*(2, 108) = 5.672, *P* = 0.005, *η*^2^ = 0.095. *Post hoc* tests showed no significant difference in accuracy between passing and the other two strategies (*Ps* > 0.05), while shooting accuracy was significantly higher than breakthrough (*P* < 0.01). A significant interaction between group and offensive strategy was observed, *F*(2, 108) = 4.657, *P* = 0.011, *η*^2^ = 0.079. The main effect of test time was significant, with Post-test accuracy (0.39 ± 0.11) significantly higher than pre-test (0.36 ± 0.11), *F*(1, 54) = 30.035, *P* < 0.001, *η*^2^ = 0.357. A significant interaction between group and test time was also found, *F*(1, 54) = 30.148, *P* < 0.001, *η*^2^ = 0.358. The interaction between decision type and offensive strategy was significant, *F*(2, 108) = 16.119, *P* < 0.001, *η*^2^ = 0.230. There was a significant three-way interaction among group, decision type, and offensive strategy, *F*(2, 108) = 4.409, *P* = 0.014, *η*^2^ = 0.075. A decision type × test time interaction was significant, *F*(1, 54) = 10.135, *P* = 0.002, *η*^2^ = 0.160, as was the group × decision type × test time interaction, *F*(1, 54) = 4.534, *P* = 0.038, *η*^2^ = 0.077. The offensive strategy × test time interaction was significant, *F*(2, 108) = 25.219, *P* < 0.001, *η*^2^ = 0.318, and the group × offensive strategy × test time interaction was also significant, *F*(2, 108) = 13.809, *P* < 0.001, *η*^2^ = 0.204. A three-way interaction among decision type, offensive strategy, and test time was significant, *F*(2, 108) = 3.136, *P* = 0.047, *η*^2^ = 0.055, as was the four-way interaction among group, decision type, offensive strategy, and test time, *F*(2, 108) = 4.679, *P* = 0.011, *η*^2^ = 0.080. No other significant main or interaction effects were found (*Ps* > 0.05).

Simple effects analyses were conducted to explore the significant interactions:

Group × Offensive Strategy: For passing, the intervention group (0.41 ± 0.12) had significantly higher accuracy than the control group (0.33 ± 0.14, *P* = 0.005); no significant differences were observed for shooting or breakthrough (*Ps* > 0.05).

Group × Test Time: At pre-test, there was no significant difference in decision accuracy between the intervention (0.36 ± 0.11) and control (0.36 ± 0.12) groups (*P* = 0.745), which indicats a comparable baseline. At Post-test, the intervention group (0.42 ± 0.10) significantly outperformed the control group (0.36 ± 0.11, *P* = 0.002). Within the intervention group, Post-test accuracy (0.42 ± 0.10) was significantly higher than pre-test (0.36 ± 0.11, *P* < 0.001), suggesting that MOT training improved decision-making ability effectively. No significant change was found in the control group (*P* = 0.994).

Decision Type × Offensive Strategy: For passing, there was no significant difference between intuitive (0.36 ± 0.12) and cognitive (0.38 ± 0.14) decisions (*P* = 0.139). For shooting and breakthrough, cognitive accuracy was significantly higher than intuitive (*Ps* < 0.01).

Group × Decision Type × Offensive Strategy: For intuitive decisions, there was no significant group differences across offensive strategies (*Ps* > 0.05). For cognitive decisions, the intervention group showed significantly higher accuracy in passing (0.43 ± 0.12) than the control group (0.33 ± 0.15, *P* < 0.001); no significant differences were found for shooting or breakthrough (*Ps* > 0.05). This indicates that MOT training significantly enhanced cognitive decision-making in passing.

Decision Type × Test Time: For intuitive decisions, no significant difference between pre-test (0.34 ± 0.11) and Post-test (0.35 ± 0.10), *P* = 0.159. For cognitive decisions, Post-test accuracy (0.44 ± 0.10) was significantly higher than pre-test (0.38 ± 0.11, *P* < 0.001), suggesting greater improvements in cognitive processing.

Group × Decision Type × Test Time: For intuitive decisions, no significant group differences at either time point (*Ps* > 0.05). For cognitive pre-test, no significant difference between intervention (0.38 ± 0.11) and control (0.39 ± 0.11), *P* = 0.443. For cognitive Post-test, intervention accuracy (0.48 ± 0.10) was significantly higher than control (0.40 ± 0.11, *P* < 0.001), confirming the effectiveness of MOT training in enhancing cognitive decision-making.

Offensive Strategy × Test Time: In passing, Post-test accuracy (0.41 ± 0.13) was significantly higher than pre-test (0.33 ± 0.13, *P* < 0.001). No significant differences were found in shooting or breakthrough (*Ps* > 0.05), indicating that passing decisions were most improved by the training.

Group × Offensive Strategy × Test Time: For passing pre-test, no significant difference between groups (intervention: 0.33 ± 0.13; control: 0.32 ± 0.13, *P* = 0.955). For passing Post-test, the intervention group (0.49 ± 0.12) showed significantly higher accuracy than the control group (0.34 ± 0.14, *P* < 0.001). No significant differences were found in shooting or breakthrough at either test time (*Ps* > 0.05), indicating MOT training primarily enhanced passing decision accuracy.

Decision Type × Offensive Strategy × Test Time: For passing pre-test, intuitive and cognitive decisions were not significantly different (both 0.42 ± 0.10, *P* = 0.397). For Post-test, cognitive decisions were significantly more accurate than intuitive in shooting and breakthrough (*Ps* < 0.05).

Group × Decision Type × Offensive Strategy × Test Time: For passing intuitive pre-test, there was no significant group difference (intervention: 0.34 ± 0.11; control: 0.33 ± 0.12, *P* = 0.676). For passing intuitive Post-test, the intervention group (0.42 ± 0.12) had significantly higher accuracy than the control group (0.35 ± 0.13, *P* = 0.024). No significant differences were found for shooting or breakthrough intuitive decisions at either test time (*Ps* > 0.05). For passing cognitive pre-test, no group difference was observed (intervention: 0.31 ± 0.13; control: 0.32 ± 0.14, *P* = 0.783). However, for passing cognitive Post-test, the intervention group (0.55 ± 0.11) significantly outperformed the control group (0.33 ± 0.16, *P* < 0.001). No significant differences were observed for shooting or breakthrough cognitive decisions (*Ps* > 0.05). These results demonstrate that the MOT intervention significantly improved both intuitive and cognitive passing decision accuracy in novice players.

### Comparison of mean fixation duration between the intervention and control groups

A repeated measures ANOVA was conducted with group (intervention group, control group) as well as the between-subjects variable, and decision type (intuitive, cognitive), offensive strategy (passing, shooting, breakthrough), while test time (pre-test, Post-test) as within-subjects variables. The dependent variable was mean fixation duration. The results are shown in Table 3.

**Table 3:** Comparison of average fixation time between intervention group and control group.

Group	Type	Time	Passing	Shooting	Breakthrough	Repeated measures ANOVA		
			*M*±*SD*	*M*±*SD*	*M*±*SD*	*F*	*P*	*η* ^2^
Intervention	Intuitive	Pre-test	408.65 ± 85.86	406.88 ± 123.09	403.45 ± 101.86			
			Post-test	340.99 ± 108.64	399.46 ± 81.45	390.53 ± 114.38			
		Cognitive	Pre-test	446.77 ± 102.98	460.98 ± 73.66	451.27 ± 75.99			
	Post-test		354.93 ± 92.51	424.65 ± 85.47	436.11 ± 98.48			
Control	Intuitive	Pre-test	390.87 ± 84.85	404.56 ± 105.20	406.23 ± 92.36			
			Post-test	397.52 ± 94.14	406.04 ± 100.11	395.68 ± 99.03			
		Cognitive	Pre-test	447.49 ± 78.18	466.36 ± 72.52	449.48 ± 71.75			
	Post-test		431.21 ± 80.53	446.47 ± 94.50	443.02 ± 93.74			
Group						1.666	0.202	0.030
Type						47.598	<0.001^**^	0.468
Group × Type						0.629	0.431	0.012
Strategy						4.274	0.016^*^	0.073
Group × Strategy						1.181	0.311	0.021
Time						7.776	0.007^**^	0.126
Group × Time						3.528	0.066	0.061
Type × Strategy						0.208	0.813	0.004
Group × Type × Strategy						0.164	0.849	0.003
Type × Time						2.594	0.113	0.046
Group × Type × Time						0.065	0.800	0.001
Strategy × Time						2.458	0.090	0.044
Group × Strategy × Time						3.170	0.046^*^	0.055
Type × Strategy × Time						0.396	0.674	0.007
Group × Type × Strategy × Time						0.005	0.995	0.000

**Notes.**

fixation time (ms)

* *P* < 0.05

** *P* < 0.01

The results showed a significant main effect of decision type on mean fixation duration. The fixation duration for intuitive decisions (395.91 ± 99.34 ms) was significantly shorter than for cognitive decisions (438.23 ± 85.96 ms), *F*(1, 54) = 47.598, *P* < 0.001, *η*^2^ = 0.468. A significant main effect of offensive strategy was also observed, *F*(2, 108) = 4.274, *P* = 0.016, *η*^2^ = 0.073. *Post hoc* comparisons revealed that fixation duration during passing (402.31 ± 93.62 ms) was marginally shorter than during shooting (426.92 ± 91.59 ms), *P* = 0.055, while no significant differences were found between breakthrough and either passing or shooting (*Ps* > 0.05). There was a significant main effect of test time: Post-test fixation duration (405.55 ± 96.77 ms) was significantly shorter than pre-test (428.58 ± 88.53 ms), *F*(1, 54) = 7.776, *P* = 0.007, *η*^2^ = 0.126. A significant three-way interaction effect among group, offensive strategy, and test time was found, *F*(2, 108) = 3.170, *P* = 0.046, *η*^2^ = 0.055. No other significant main effects or interaction effects were observed (*Ps* > 0.05).

To further explore the significant interaction among group, offensive strategy, and test time, simple effects analysis showed no significant group differences in fixation duration during pre-test for passing, shooting, and breakthrough (*Ps* > 0.05). However, in the Post-test passing condition, the intervention group exhibited significantly shorter fixation durations (347.97 ± 100.58 ms) compared to the control group (414.36 ± 87.33 ms), *P* = 0.002. This suggests that while both groups had similar gaze behaviors at baseline, integrating MOT training into basketball instruction led to a more pronounced reduction in fixation duration during passing.

### Comparison of mean number of fixations between the intervention and control groups

A repeated measures ANOVA was conducted with group (intervention, control) whlie the between-subjects factor, and decision type (intuitive, cognitive), offensive strategy (passing, shooting, breakthrough), and test time (pre-test, Post-test) as within-subjects factors. The dependent variable was the mean number of fixations, and the results are presented in Table 4. The analysis revealed a significant main effect of decision type: intuitive decisions (7.98 ± 2.32) were associated with significantly fewer fixations than cognitive decisions (8.27 ± 2.10), *F*(1, 54) = 5.180, *P* = 0.027, *η*^2^ = 0.088. There was also a significant main effect of test time: Post-test fixations (7.83 ± 2.33) were significantly fewer than pre-test (8.41 ± 2.09), *F*(1, 54) = 5.888, *P* = 0.019, *η*^2^ = 0.098. No other significant main effects or interaction effects were found (*Ps* > 0.05).

**Table 4:** Comparison of average fixation frequency between intervention group and control group.

Group	Type	Time	Passing	Shooting	Breakthrough	Repeated measures ANOVA		
			*M*±*SD*	*M*±*SD*	*M*±*SD*	*F*	*P*	*η* ^2^
Intervention	Intuitive	Pre-test	8.24 ± 2.22	8.21 ± 1.76	8.21 ± 2.16			
			Post-test	7.12 ± 2.47	7.77 ± 2.81	7.68 ± 2.81			
		Cognitive	Pre-test	8.63 ± 2.74	8.70 ± 1.92	8.50 ± 1.54			
	Post-test		7.23 ± 1.30	7.83 ± 2.18	7.66 ± 2.33			
control	Intuitive	Pre-test	8.32 ± 2.21	8.20 ± 2.19	7.98 ± 1.96			
			Post-test	8.06 ± 2.45	8.04 ± 2.50	7.88 ± 2.50			
		Cognitive	Pre-test	8.78 ± 2.25	8.61 ± 2.45	8.52 ± 1.85			
	Post-test		8.31 ± 1.78	8.22 ± 2.18	8.18 ± 2.54			
Group						0.920	0.342	0.017
Type						5.180	0.027^*^	0.088
Group × Type						0.288	0.594	0.005
Strategy						0.351	0.705	0.006
Group × Strategy						1.144	0.322	0.021
Time						5.888	0.019^*^	0.098
Group × Time						1.489	0.228	0.027
Type × Strategy						0.004	0.996	0.000
Group × Type × Strategy						0.080	0.923	0.001
Type × Time						0.583	0.449	0.011
Group × Type × Time						0.022	0.882	0.000
Strategy × Time						0.536	0.586	0.010
Group × Strategy × Time						0.245	0.783	0.005
Type × Strategy × Time						0.007	0.993	0.000
Group × Type × Strategy × Time						0.006	0.994	0.000

**Notes.**

* *P* < 0.05

### ERP Characteristics

**Figure 4:**
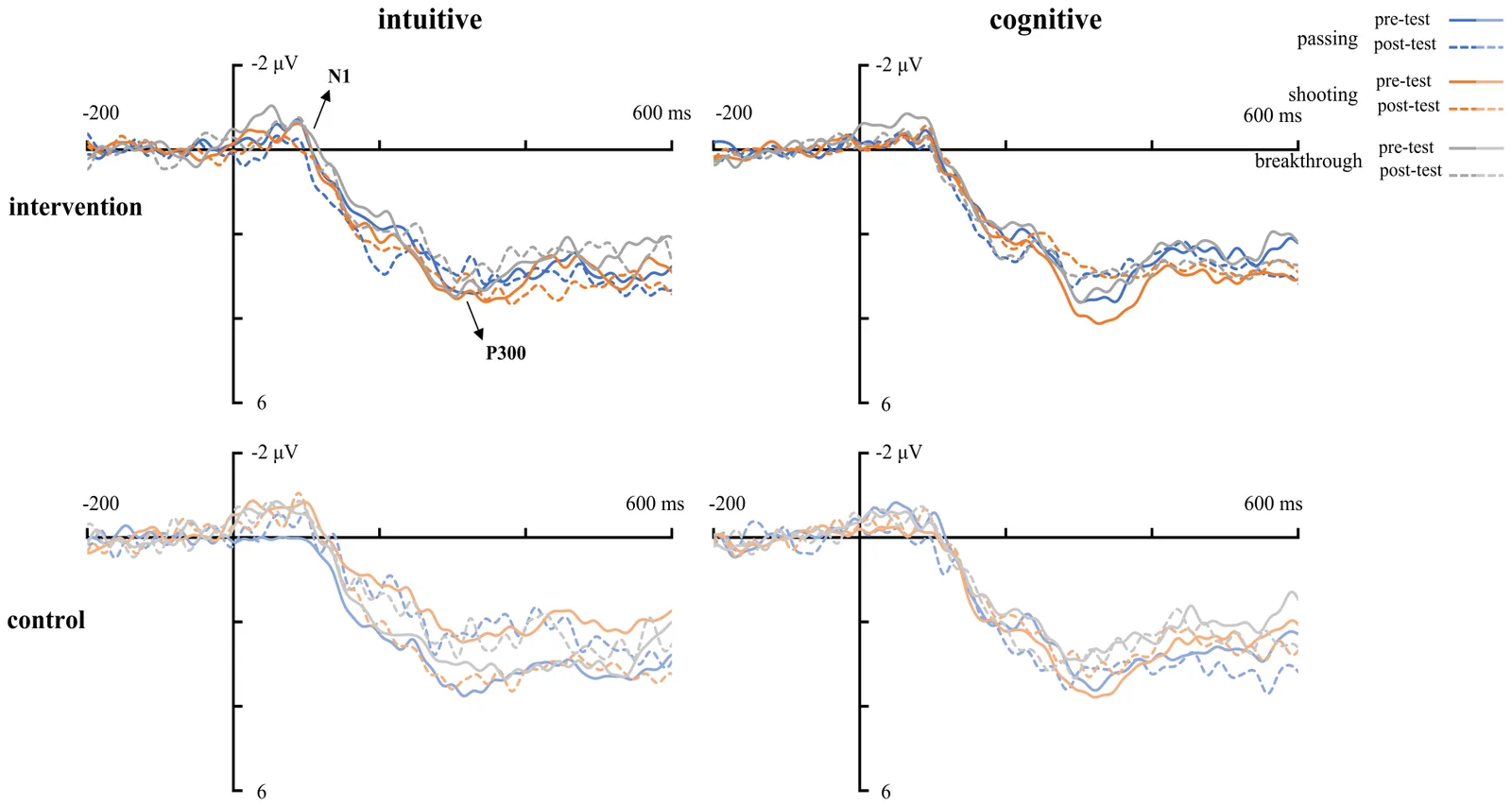
ERP waveform of the central parietal region after video in intervention group and control group.

**Figure 5:**
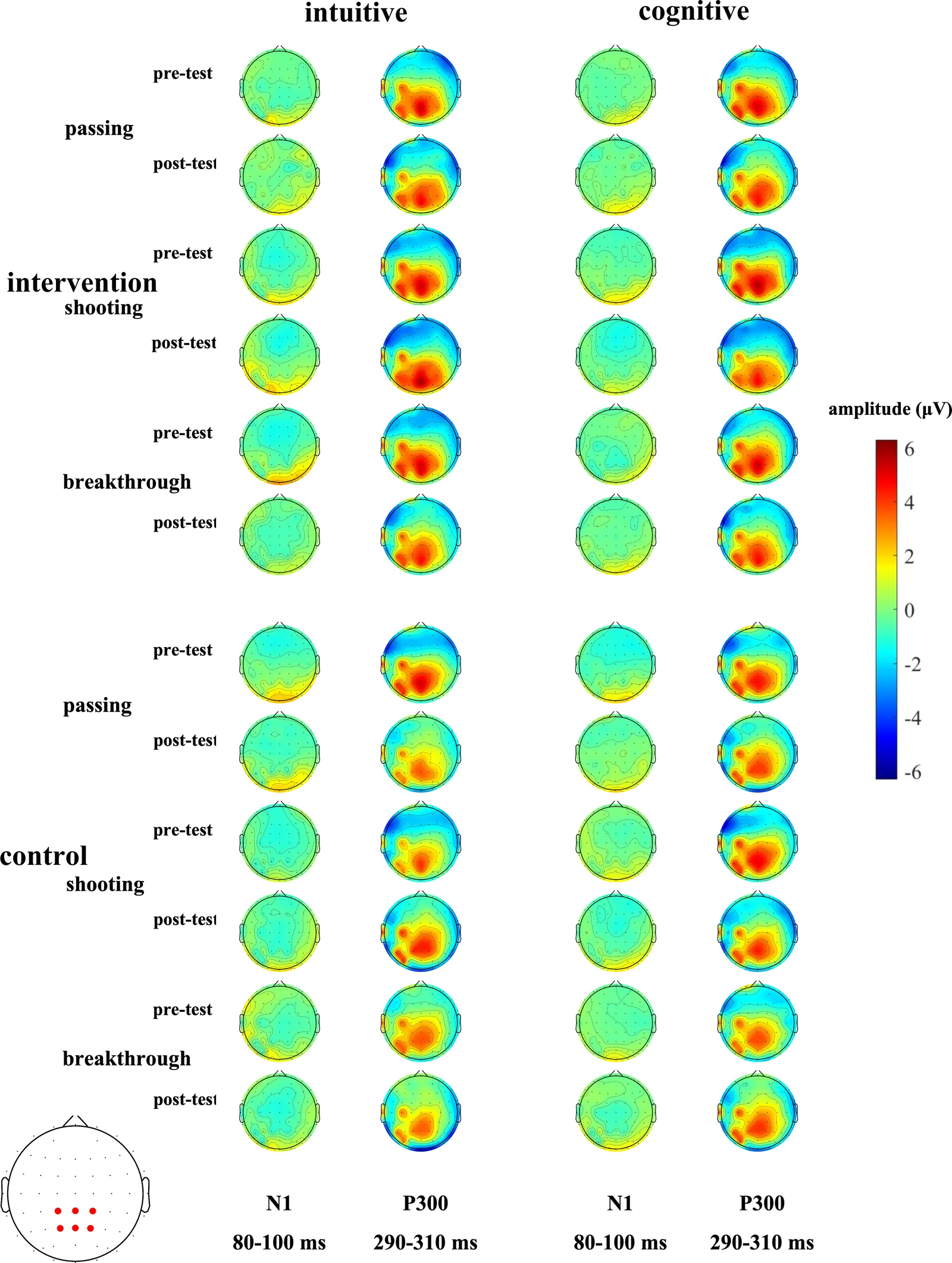
Topographic map of the central parietal region in intervention group control group after video.

The ERP waveform plots, brain topographic maps, and amplitude analyses were conducted based on the temporal progression of the video stimulus presentation, categorized into early, late, and end phases. Taking the ERP analysis after the video ended as an example: The ERP waveforms evoked at the centro-parietal electrodes in the intervention and control groups following the video offset are shown in Fig. 4. In the figure, 0 ms represents the time point at which the video ended. The corresponding brain topographic maps are presented in Fig. 5.

 The results indicated that both the intervention and control group players elicited two distinct ERP components after the video ended under varying conditions of decision type, offensive strategy, and test time. Specifically, a negative-going N1 component peaked between 80–100 ms, and a positive-going P300 component peaked between 290–310 ms. The N1 component exhibited negative amplitude values, with more negative amplitudes indicating greater component magnitude. In contrast, the P300 component showed positive amplitude values, with more than positive amplitudes reflecting greater magnitude. Both components reached their maximum amplitudes at the centro-parietal electrode sites. Therefore, six electrodes in this region (CP1, CPz, CP2, PO1, POz, and PO2) and the corresponding time windows were selected for further analysis of these two components.

A repeated measures ANOVA was conducted with group (intervention group, control group) as the between-subjects factor, and decision type (intuitive, cognitive), attack style (passing, shooting, breakthrough), and test time (pre-test, Post-test) as within-subjects factors. The dependent variables were the amplitudes of the N1 and P300 components recorded at six central-parietal to parieto-occipital electrodes (CP1, CPz, CP2, PO1, POz, and PO2) after the end of the video stimuli.

For the N1 component, the main effect of decision type was significant. The N1 amplitude under the cognitive decision condition (−0.32 ± 0.23 μV) was significantly smaller than that under the intuitive decision condition (−0.50 ± 0.30 μV), *F*(1, 54) = 4.329, *P* = 0.042, *η*^2^ = 0.074. The main effect of attack style was also significant, *F*(2, 108) = 4.444, *P* = 0.014, *η*^2^ = 0.076. *Post hoc* comparisons showed that the N1 amplitude for passing was significantly smaller than that for shooting and breakthrough (*Ps* < 0.05), while there was no significant difference between shooting (−0.46 ± 0.29 μV) and breakthrough (−0.55 ± 0.24 μV), *P* = 0.375. This indicates that the N1 amplitude was smallest for passing. No other significant main or interaction effects were found (*Ps* ≥ 0.130).

For the P300 component, none of the main effects were significant (*Ps* ≥ 0.209). However, the interaction among decision type, attack style, and test time was significant, *F*(2, 108) = 3.904, *P* = 0.023, *η*^2^ = 0.067. Simple effects analysis further revealed a marginally significant decrease in P300 amplitude for shooting under the cognitive decision condition from pre-test (3.76 ± 0.46 μV) to Post-test (2.81 ± 0.51 μV), *P* = 0.061. No significant differences in P300 amplitudes were observed between pre-test and Post-test for passing or breakthrough under the cognitive decision condition, nor for any attack style under the intuitive decision condition (*Ps* ≥ 0.119), indicating a specific reduction in P300 amplitude for shooting in the cognitive condition after training. No other significant interaction effects were found (*Ps* ≥ 0.288). See Table 5.

**Table 5:** Comparison of wave amplitudes of various components in the central parietal region between two groups after video.

Component	Group	Type	Time	Passing	Shooting	Breakthrough
				*M*±*SE*	*M*±*SE*	*M*±*SE*
N1 (μV)	Intervention	Intuitive	Pre-test	−0.59 ± 0.20	−0.56 ± 0.41	−0.61 ± 0.24
					Post-test	−0.14 ± 0.36	−0.02 ± 0.31	−0.66 ± 0.30
				Cognitive	Pre-test	−0.37 ± 0.30	−0.24 ± 0.19	−0.69 ± 0.21
			Post-test		−0.05 ± 0.25	−0.47 ± 0.25	−0.21 ± 0.19
		Control	Intuitive	Pre-test	0.03 ± 0.20	−0.78 ± 0.41	−0.57 ± 0.24
					Post-test	−0.41 ± 0.36	−0.90 ± 0.31	−0.76 ± 0.30
				Cognitive	Pre-test	−0.37 ± 0.30	−0.13 ± 0.19	−0.28 ± 0.21
	Post-test		0.06 ± 0.25		−0.55 ± 0.25	−0.62 ± 0.19
P300 (μV)	Intervention	Intuitive	Pre-test	3.31 ± 0.41	3.46 ± 0.52	3.40 ± 0.52
					Post-test	2.91 ± 0.60	3.23 ± 0.55	2.95 ± 0.70
				Cognitive	Pre-test	3.55 ± 0.35	3.89 ± 0.46	3.54 ± 0.43
			Post-test		3.11 ± 0.55	2.70 ± 0.51	3.01 ± 0.55
		Control	Intuitive	Pre-test	3.55 ± 0.41	2.29 ± 0.52	2.99 ± 0.52
					Post-test	2.32 ± 0.60	3.53 ± 0.55	2.63 ± 0.70
				Cognitive	Pre-test	3.35 ± 0.35	3.62 ± 0.46	2.90 ± 0.43
	Post-test		3.22 ± 0.55		2.92 ± 0.51	3.15 ± 0.55

## Discussion

The results of this study generally support H1–H3: MOT training significantly improved decision-making performance (faster reaction time and higher accuracy), optimized visual search strategies (shorter and more focused fixations), and modulated ERP components (P1, N1, LPC, and LNC), reflecting enhanced neural efficiency. These findings indicate that MOT training promotes multi-level improvements in sports decision-making by enhancing attentional allocation and information processing efficiency. Early research emphasized that professional executors often exhibit longer focus on task related clues, reflecting more effective information acquisition rather than superficial scanning (Gou & Li, 2025). This indicates that the relationship between gaze behavior and superior decision-making is subtle and dependent on the context.

These findings are consistent with those of Romeas, Guldner & Faubert (2016), who demonstrated that 3D-MOT training significantly improved the accuracy of passing decisions in soccer players, which suggesting that perceptual–cognitive training has a positive transfer effect on in-game decision-making. While the present study demonstrated improvements in visual attention and passing-related decision-making among novice players, previous research has raised important qualifications. Vater, Gray & Holcombe (2021) argued that MOT transfer was not universal and likely depends on the degree of overlap between the training task and the target performance domain. For supporting this view, Harenberg et al. (2022) observed no measurable gains in game-specific decision-making after MOT training in youth players, suggesting that perceptual–cognitive improvements may not automatically translate to complex tactical behaviors. Similarly, Romeas et al. (2025) reported enhanced attentional metrics but limited transfer to actual sport performance, emphasizing that training effects may be constrained by task specificity and skill level. By contrast, our novice sample—characterized by lower baseline attentional efficiency—may have been more sensitive to improvements induced by MOT training, particularly in passing contexts that rely heavily on peripheral vision and rapid target selection. Integrating these findings, MOT training may offer targeted benefits, but its transferability is selective rather than general.

Transfer from computer-based perceptual training to on-court performance is not automatic. From a theoretical perspective, MOT is designed to train domain-general perceptual–cognitive functions (*e.g.*, divided attention, dynamic attentional allocation, and multiple object monitoring) that are fundamental to team sports. Basketball performance requires continuous tracking of multiple teammates and opponents, rapid updating of spatial relations, and timely action selection. Thus, MOT shares key functional demands with in-game perceptual processing, even though the surface features differ. Importantly, we acknowledge the Modified Perceptual Training Framework (Hadlow et al., 2018),which emphasizes that transfer depends on the degree of functional and informational overlap between training and performance contexts. We clarify that the observed transfer in our study was selective (*i.e.,* primarily in passing decisions), rather than global. This pattern is consistent with the framework’ s prediction that partial overlap yields partial transfer.

One of the cognitive mechanisms underlying decision-making is the visual search strategy, which serves as a crucial pathway for understanding athletic decision-making (Vaeyens et al., 2007). Researchers have employed the expert-novice paradigm to explore differences in cognitive processing among players, and eye-tracking technology has provided new methods for evaluating visual attention in sports contexts (Liu & Tang, 2022). Elite players often adopt context-specific visual strategies to optimize decision-making (Savelsbergh et al., 2010). For instance, padel players focus on the ball and the upper body of opponents during interception actions (Palma, Del Campo & Marín, 2023). During decision-making, players must concentrate attention on critical environmental stimuli, integrate this input with prior experience, and respond rapidly (Suárez et al., 2020). Improvements in visual search behavior were also reflected in reduced fixation duration and pupil diameter within short time windows. Changes in pupil size are believed to be influenced by the interplay of emotional arousal and cognitive empathy elicited by the video stimuli (Zhang et al., 2023). The performance of the parafoveal region plays a crucial role in visual cognition and perceptual processes (Klostermann et al., 2020). Players with superior decision-making abilities tend to exhibit more fixation points and shorter fixation durations in complex scenes involving multiple players (Fortes et al., 2021). Brief fixations allow players to perceive player positions, movements, and optimal passing opportunities (Afonso, Garganta & Mesquita, 2012).

The analysis of ERP waveforms, topographical maps, and amplitude measurements further supports our hypothesis. In the Post-test, the intervention group demonstrated significantly higher accuracy rates in passing decisions compared to the control group (*P* < 0.01). During the early phase of the video stimuli, ERP components N1, P1, and N250 peaked at fronto-central electrodes, while P1, N1, and P300 peaked at parieto-occipital electrodes. The P1 component reflects spatial discrimination of visual stimuli and is related to the allocation of attentional resources (Luck et al., 1994), while N1 was associated with recognition or discrimination of stimulus features and is indicative of selective attention (Hillyard, Vogel & Luck, 1998). The P1 and N1 components predominantly occurred in fronto-central and parieto-occipital areas, which aligns with findings by Liu (2016). MOT training resulted in reduced N1 amplitudes for passing in the intervention group, and significantly smaller P1 amplitudes compared to the control group (*P* < 0.05). This suggests that MOT training enabled novice players to allocate attentional resources more efficiently in the early stages of stimulus presentation, with more targeted selective attention. In contrast, players without MOT training deployed greater cognitive resources early on. This implies that during the initial phase of visual presentation—when task difficulty is high—players must quickly search for offensive player positioning, allocating substantial cognitive resources to “reading the game”. No significant differences were found between groups in the N250 and P300 components (*Ps* > 0.05).

In the later phase of the video, the LPC and LNC components peaked in the fronto-central and parieto-occipital areas. Prior to decision execution, the prominent LNC component indicated that players engaged in information processing-recognition, analogical reasoning, and decision-making based on selective attention to key visual cues presented earlier. This also explains the stronger whole-brain activation observed in the topographic maps during the 1,800–3,000 ms interval. MOT training led to significantly increased LNC amplitude in the fronto-central region (*P* < 0.05) and significantly reduced LPC amplitude in the parieto-occipital region (*P* < 0.05) for the intervention group. This suggests that trained players allocated more cognitive resources to processing critical decision cues and identifying optimal timing-findings consistent with Zhang & Zhou (2013). LPC was associated with rehearsal in working memory, whereas LNC may serve as a marker of memory storage (Werrmann & Niedeggen, 2023). MOT training improved visual attention in the intervention group and facilitated the encoding of relevant tracking information, enabling players to quickly recognize teammates and game situations. Eye-tracking data further revealed that the intervention group’s attention was concentrated on key and relevant regions of the screen.

After the video ended, N1 and P300 components peaked in the centro-parietal region as participants were prompted to respond *via* keypress. No significant group differences were found in these components (*Ps* > 0.05), which suggesting that by the end of the video, players were cognitively prepared for decision execution. Participants’ attentional resources were primarily allocated to the target stimulus, with longer fixations on targets compared to non-targets. EEG potentials associated with eye fixation are difficult to interpret before the target was identified, due to challenges in tracking the temporal dynamics of attention allocation—an issue that eye-tracking technology can resolve. In traditional ERP paradigms, participants typically do not respond to non-target stimuli, limiting researchers’ ability to assess attentional distribution prior to target recognition. Moreover, EEG data is often contaminated by ocular artifacts, which is why ERP experiments generally require participants to limit eye movements. Such as conventional ERP studies are not suitable for investigating visual search in naturalistic settings. However, the synchronized EyeLink-EEG technique enables the study of attention allocation under free-viewing conditions. Fixation-related potentials (EFRPs) reflect brain processes synchronized with visual information input and allow researchers to examine attention distribution among non-targets without requiring motor responses (Fuseda et al., 2021). EFRPs and ERPs essentially reflect the same underlying neural activity (Yagi, 1995). The observed reduction in reaction time and the power during visual search tasks in players compared to non-players supports the neural efficiency hypothesis, suggesting that cognitive improvement results from shorter latencies and decreased beta power (Vicente et al., 2023).

Several limitations should be considered when interpreting the present findings. First, the study was conducted primarily in a laboratory-based setting using screen based tasks, which may limit ecological validity and the generalizability of the results to real world basketball performance. Improvements in “correct” decisions on a video-based task do not automatically equate to becoming a “better player” in a holistic sense. The display size and resolution were also relatively modest compared with current technological standards, potentially constraining perceptual realism. Future studies should employ larger displays, immersive environments, or *in situ* testing to better approximate competitive conditions (Zhang et al., 2024). Second, the sample consisted exclusively of novice female basketball players. While this population may be particularly sensitive to perceptual–cognitive training, the findings may not generalize to more experienced players or other populations. Replication across different skill levels and competitive contexts is therefore warranted. Third, although the MOT training task and the video-based decision-making measures were designed in accordance with commonly used paradigms in perceptual–cognitive and SDM research, they have not yet undergone formal psychometric validation. This limitation constrains the strength of inferences regarding construct validity, and future research should establish the reliability and validity of these measures more rigorously. Fourth, transfer effects of MOT training remain debated in the literature, with many studies reporting null or inconsistent findings. In the present study, training-related improvements were selective—primarily observed in passing decisions among novice players—rather than generalized across all decision types. This pattern suggests population and task-specific sensitivity rather than broad transfer and underscores the need for cautious interpretation. Longitudinal designs and larger samples are needed to determine the robustness and durability of these effects. Finally, although the findings provide preliminary support for incorporating visuocognitive assessments and training into talent development and coaching practice, integrating MOT training into routine sport-specific training environments may yield more informative insights into its potential impact on in-game performance and broader cognitive functions. From an applied perspective, MOT training may serve as an effective supplement to traditional coaching by enhancing players’ attentional allocation and decision-making efficiency. Integrating perceptual–cognitive training with sport-specific drills may further facilitate transfer to real-game performance. Future training programs should consider incorporating adaptive visuocognitive tasks and individualized feedback mechanisms to optimize learning outcomes.

##  Supplemental Information

10.7717/peerj.21711/supp-1 Supplemental Information 1 Raw data

10.7717/peerj.21711/supp-2 Supplemental Information 2 Validity of stimulus materials

## Additional Information and Declarations

### Data Availability

The following information was supplied regarding data availability: The raw measurements are available in the Supplemental Files.
