# Supplementary material for: Effects of multiple object tracking training on visual attention and sports decision-making in novice female basketball players: evidence from EyeLink-EEG synchronization
Source: PeerJ. 2026 Sep 9;14:e21711. doi: 10.7717/peerj.21711 (PMC13570609; doi:10.7717/peerj.21711)
Supplement: Supplementary file 1 [file peerj-14-21711-s002.pdf]

Validity of stimulus materials

1 Determination of content validity

Based on the content validity ratio (CVR) and content validity index (CVI) methods proposed by Lawshe, the content validity was determined through evaluation by five basketball experts,

(1) Inclusion criteria of the expert group

3 Chinese University Basketball League (CUBAL) basketball head coaches: B-level coaches with senior professional titles, who have been engaged in basketball teaching or training for over 10 years; 2 Chinese Basketball Association (CBA) players: both are national-level competition winners, with over 10 years of professional basketball training, excellent basketball skills, and rich competition experience.

(2) Evaluation method

Using the four-part evaluation method proposed by Lawshe to assess video materials (Lawshe, 1975), it encompasses the following four dimensions: ①Task matching: whether it conforms to the definitions and technical requirements of passing, shooting, and breakthrough offensive methods; ②Situational authenticity: whether it closely resembles actual basketball game scenarios; ③ Technical normativity: whether it adheres to the technical norms of basketball-specific movements; ④Difficulty rationality: whether it is suitable for the ability level of the experimental subjects.

Based on professional knowledge and experience, basketball experts comprehensively evaluate each video. The Likert 4-point scoring method is used to assess the content validity of the decision-making videos: 1 indicates "completely does not match", 2 indicates "somewhat matches but requires significant improvement", 3 indicates "matches but requires minor improvement", and 4 indicates "completely matches".

(3) Data processing

Calculate the proportion of five experts who rate each video as "3" or "4", and compute the internal score of a single video

Item-level content validity index (I-CVI);

Calculate the average of all video I-CVI values to obtain the overall content validity index (scale-level content)

validity index, S-CVI) (Lu et al., 2024);

③ Referring to the research conducted by Polit and Beck, a content validity of 0.8 is considered as the acceptable standard (Polit et al., 2006).

(4) Verification results

Table 1 Content validity assessment of experimental video materials

Offensive Strategy	Number of videos	I-CVI	Average I-CVI	Overall S-CVI	Evaluation results
passing	40	0.8-1	0.84	0.86	qualified
shooting	40	0.8-1	0.89		qualified
breakthrough	40	0.8-1	0.86		qualified

The results showed that the I-CVI of the 120 videos ranged from 0.8 to 1, with the average I-CVI for passing, shooting, and penetration videos being

All values are greater than 0.8, and the overall S-CVI of the videos for the three offensive methods is 0.86, indicating high content validity. This can fully represent basketball-specific tasks and ensure the scientific and rigorous nature of the experimental design. See Table 1.

2. Determination of construct validity

The study additionally selected 6 high-level basketball players and 6 college students taking basketball as an elective course for validity testing. The aim is to eliminate the "ceiling effect" and "floor effect" to ensure that each video stimulus material can effectively distinguish the decision-making efficiency of athletes of different levels. For videos with low differentiation, they are edited again by extending or shortening the video duration until each video can effectively distinguish the differences in decision-making agility and rationality between high-level athletes and college students in elective courses, that is, high-level athletes have higher decision-making speed and accuracy(Dai et al., 2011).

References

Lawshe, C. (1975). A quantitative approach to content validity. *Personnel Psychology*, 28(4):563-575.https://doi.org/10.1111/j.1744-6570.1975.tb01393.x

Lu, J., Shi, L., Qiu, C., Wang, F., Li, X., Pan, S., Zhao, J., Yan, S., & Jin, Z. (2024). The reliability and validity of the Chinese version of the visually induced motion sickness susceptibility questionnaire, *Journal of Audiology and Speech Pathology*, 32(3):193-199.https://doi.org/10.3969/j.issn.1006-7299.2024.03.001

Polit, D., & Beck, C.(2006). The content validity index: are you sure you know what's being reported? Critique and recommendations. *Research in Nursing and Health*, 29(5): 489-497.https://doi.org/10.1002/nur.20147

Dai, H., Zhang, F., & Chen, X.(2011).*Psychological and Educational Measurement.* Jinan University Press.
